# Cannabidiol and Terpene Formulation Reducing SARS-CoV-2 Infectivity Tackling a Therapeutic Strategy

**DOI:** 10.3389/fimmu.2022.841459

**Published:** 2022-02-15

**Authors:** Susana Santos, Pedro Barata, Adilia Charmier, Inês Lehmann, Suzilaine Rodrigues, Matteo M. Melosini, Patrick J. Pais, André P. Sousa, Catarina Teixeira, Inês Santos, Ana Catarina Rocha, Pilar Baylina, Ruben Fernandes

**Affiliations:** ^1^ R&D&Innovation Department, EXMceuticals Portugal Lda, Lisboa, Portugal; ^2^ Cooperativa de Formação e Animação Cultural – Centre for Interdisciplinary Development and Research on Environment, Applied Management and Space (COFAC-DREAMS)-Universidade Lusófona, Lisboa, Portugal; ^3^ LABMI – Laboratório de Biotecnologia Médica e Industrial, PORTIC – Porto Research, Technology and Innovation Center, Porto, Portugal; ^4^ Metabesity Deopartment, i3S – Instituto de Investigação e Inovação em Saúde, Porto, Portugal; ^5^ Escola Superior de Saúde, Instituto Politécnico do Porto, Porto, Portugal

**Keywords:** CBD - cannabidiol, endocannabinoid system (ECS), SARS-CoV-2, therapeutics, terpenes, formulations, essential oil (EO)

## Abstract

In late 2019, COVID-19 emerged in Wuhan, China. Currently, it is an ongoing global health threat stressing the need for therapeutic compounds. Linking the virus life cycle and its interaction with cell receptors and internal cellular machinery is key to developing therapies based on the control of infectivity and inflammation. In this framework, we evaluate the combination of cannabidiol (CBD), as an anti-inflammatory molecule, and terpenes, by their anti-microbiological properties, in reducing SARS-CoV-2 infectivity. Our group settled six formulations combining CBD and terpenes purified from *Cannabis sativa* L, *Origanum vulgare*, and *Thymus mastichina*. The formulations were analyzed by HPLC and GC-MS and evaluated for virucide and antiviral potential by *in vitro* studies in alveolar basal epithelial, colon, kidney, and keratinocyte human cell lines.

**Conclusions and Impact:**

We demonstrate the virucide effectiveness of CBD and terpene-based formulations. F2TC reduces the infectivity by 17%, 24%, and 99% for CaCo-2, HaCat, and A549, respectively, and F1TC by 43%, 37%, and 29% for Hek293T, HaCaT, and Caco-2, respectively. To the best of our knowledge, this is the first approach that tackles the combination of CBD with a specific group of terpenes against SARS-CoV-2 in different cell lines. The differential effectiveness of formulations according to the cell line can be relevant to understanding the pattern of virus infectivity and the host inflammation response, and lead to new therapeutic strategies.

## 1 Introduction

Since the emergence of the SARS-CoV-2 outbreak, extensive efforts have been placed regarding antiviral research for compounds with effective antiviral or virucide activity. COVID-19 is a complex disease that afflicts respiratory and gastrointestinal tract and kidney function, being one of its main features the hyperstimulation of the immune system. The spectrum of medical therapies to treat COVID-19 is growing; however, there are no 100% effective therapeutic approaches for the prevention and treatment of COVID-19.

### 1.1 Cannabis, Origanum, and Thymus Species as a Source of Biobased Formulations for Limiting SARS-CoV-2 Infectivity

Although vaccination and preventive medications are recognized as the most effective means of combating a virus, the treatment of COVID-19 is a real challenge prompting the need for effective drugs (1). Natural compounds from medicinal plants, such as terpenes, have gained attention as potential inhibitors of coronaviruses being the possible mechanism in the inhibition of viral replication or targeting viral proteins relevant for virus adsorption and entry (1–7). Essential oils (EOs) exhibit antiviral (4, 8–12), immunomodulatory (8, 13), and anti-inflammatory (8, 14, 15) properties, namely, regarding virus infection as *influenza* or herpes simplex viruses 1 or 2 (16, 17). EOs from *Origanum acutidens *(18), *Artemisia glabella *(19), eucalyptus and tea tree (20), *Thymus vulgaris*, *Melaleuca ericifolia*, *M*. *leucadendron*, and *M. armillaris* (21), among many others, have been described against those viruses. Wen et al. (22) reported EO constituents inducing a cytopathogenic effect against SARS-CoV on Vero-E6 cells. From molecular modeling studies, several terpenoids were potent inhibitors of SARS-CoV-2 replication (23). Among medicinal plants, *Origanum vulgare* has been recognized for its potential therapeutic role mainly arising from terpenes and flavonoids (24, 25) and *Thymus vulgaris* EOs have been shown to be effective against several RNA viruses including coronaviruses (21, 26). Regarding *Cannabis sativa*, it is particularly rich in terpenes, typically monoterpenes and sesquiterpenes (27–30). This plant is mostly known for containing phytocannabinoids, mainly Δ^9^-tetrahydrocannabinol (THC) and cannabidiol (CBD), those widely accessed being medicinal compounds with known applications in several conditions, most of them related to inflammation processes (30–36). Phytocannabinoids are a group of terpenophenolic compounds with biological activities through interaction with the endocannabinoid system (ECS) in humans. CBD is a partial agonist for cannabinoid receptor 2 (CN2R), widely expressed in the immune system (37–39). In a mouse model for a respiratory syncytial virus (RSV) infection, CN2R activation reduced the signs of infection by modulating the immune system (10). One study demonstrates that a genetic polymorphism in CN2R, which reduces ECS-induced response (40), is associated with an increased risk of hospitalization in young children infected with RSV (n = 83), with up to 3-fold increased risk of developing severe acute respiratory tract infection. Rossi et al. (41) hypothesize that CN2R can be a therapeutic target for SARS-CoV-2 since its stimulation limits the release of pro-inflammatory cytokines, shifts the macrophage phenotype toward the anti-inflammatory M2 phenotype, and enhances the immune-modulating properties of mesenchymal stromal cells. The anti-inflammatory properties of CBD have been explored as antiviral agents for the treatment of HIV (42), *influenza* (43, 44), and most recently SARS-CoV-2 (45–48). CN2R increases as HIV infection progresses, and on infected macrophages, the exposure to CN2R to a selective agonist resulted in a dose-dependent decrease in reverse transcriptase activity/viral replication activity (17). Recently, researchers have tested CBD on 3D human models of oral, airways, and intestinal tissues and found that low THC/high CBD cultivars modulate ACE2 and TMPRSS2 levels, which might lower the virus load (49). By its turn, another study demonstrated that CBD reduced the secretion of pro-inflammatory cytokines IL-6, IL-8, CCL2, and CCL7 from the alveolar epithelial cell line A549 (50).

### 1.2 Why Does a Trade of *Two* Make Sense for Opposing One Single Agent? The Rationale for the Formulations

A similar study to the one proposed in this work was executed by Chatow et al. (51) who demonstrated an antiviral activity of a terpene formulation (30 terpenes) against HCoV-229E in human lung fibroblasts and the antiviral action during the viral multiplication cycle, in which the combination of the CBD with terpenes potentiated the antiviral effect. In another study, CBD exerted prolonged immunosuppression and hence might be used in chronic inflammation, and the terpenoids showed transient immunosuppression and might thus be used to relieve acute inflammation (52). Since terpenes are known to act as enhancing phytocannabinoid action (53–55), it is intended to query the action of a group of specific terpenes as virucide or antiviral agents acting in entourage effect with their selves and with the CBD as a therapeutic agent modulating inflammation. In detail, to exploit the biological action of the formulations on virus infectivity, it is a goal to understand their potential effect to act i) as a virucide agent blocking and inactivating the virus at early stages of infection, ii) as an antiviral agent blocking the virus cellular machinery, iii) as an agent against an overactive immune-inflammatory cascade at later stages of infection. This could be relevant in reversing the cytotoxic events induced by the virus and may contribute to the concept of ECS as a contributor for controlling the immune response from a virus infection.

## 2 Materials and Methods

### 2.1 Formulation Development and Analysis

CBD and terpenes identified from *Cannabis sativa*, *Origanum vulgare subsp. virens*, and *Thymus mastichina* provided the source for 6 proprietary formulations (F1T, F2T, F3T, F1TC, F2TC, F3TC). The CBD was purified from a *Cannabis sativa* distillate (FarmCeutica Wellness, Richmond, Canada). A purified CBD sample (>99.5%) was obtained by Centrifuge Partition Chromatography (CPC) technology using an rCPC device (RotaChrom, Purified Solutions, Budapest, Hungary). The method was internally developed through optimizing the best solvent combination and the solvent ratio (data to be published). Briefly, CPC is a liquid–liquid preparative chromatographic technique that makes use of two immiscible liquid phases, the solvent system, representing the stationary and mobile phases of a typical chromatographic apparatus. The target terpenes of EOs and CBD from the distillate were separated according to their partition coefficients. The main goal of this technology is to isolate specific compounds with a pharmaceutical purity grade (>99%) and high recovery mass yield (>95%). Relative to terpene purification, 3 groups of specific terpenes (19 in total) were purified from *Origanum vulgare subsp. virens* and *Thymus mastichina* (NBI-Natural Business Intelligence, Vila Real, Portugal) and from *Cannabis sativa* by combining a hydrodistillation clevenger with CPC (proprietary method). The formulations F1T, F2T, and F3T were prepared to contain a specific group of 7, 8, and 9 terpenes, respectively, comprising approximately 84% of terpene content and using MCT oil as carrier oil. The remaining terpenes were present at concentrations smaller than 2%. Moreover, CBD was added at a concentration of 1 µg/ml intending formulations F1TC, F2TC, and F3TC. Formulations and CBD samples were kept in dark amber glass flasks at room temperature. The EOs were obtained by the hydrodistillation clevenger, and the formulations comprising the terpenes were analyzed by gas chromatography-mass spectrometry (GC-MS, GC-MS-QP2020 NX Gas Chromatograph Mass Spectrometer, Shimadzu, Kyoto, Japan) (**Figures S1A–C** and **Supplemental Information**). The CBD isolate was analyzed by high-performance liquid chromatography (HPLC, Cannabis Analyzer™ for Potency, Shimadzu) (**Figure S1D** and **Supplemental Information**).

### 2.2 *In Vitro* Virucide Assays

#### 2.2.1 Cell Culture

The Caco-2 cell line was cultured in MEM medium (VWR, Biowest, P0451-N10L, Riverside, MO, USA) supplemented with 20% of FBS (Gibco, Life Technologies, 10270, Grand Island, NY, USA) and 1% penicillin/streptomycin (Gibco, Life Technologies, 10270, USA). A549, HaCaT, and Hek293T cell lines were cultured in DMEM medium (VWR, Biowest, P0103-N10L, USA) supplemented with 10% of FBS and 1% penicillin/streptomycin. Cells were maintained at 37°C in a humidified chamber containing 5% CO_2_.

#### 2.2.2 SARS-CoV-2 Expansion

The B.1.1.7 strain of SARS-CoV-2, isolated in the laboratory, was clarified by centrifugation at 2,000 g for 15 min. The isolated virus was incubated in each cell line with 2% trypsin for 1 h, and then the cell culture was washed twice with PBS and incubated with complete cell culture medium.

#### 2.2.3 Determination of the SARS-CoV-2 Titer

Total RNA was extracted using Lab-Aid Virus RNA Extraction Kit (Zeesan, Xiamen, China). RNA purity was measured in a microdrop 16-well microplate spectrophotometer (Thermo Scientific™ Multiskan SkyHigh Microplate Spectrophotometer, Life Technologies, Thermo Fisher Scientific, Waltham, MA, USA). Virus titer was determined by SARS-CoV-2 detection with Fosun COVID-19 RT-PCR Detection Kit (Fosun Pharma, Shanghai, China) and quantified using a calibration curve with Synthetic SARS-CoV-2 RNA SARS-CoV-2 positive control (SARS-CoV-2 Positive Control, Twist Synthetic, China).

#### 2.2.4 Cytotoxicity Evaluation of Formulations

The MTT assay (Life Technologies, Thermo Fisher Scientific, USA) was carried out following the manufacturer’s instruction. In brief, 1 × 10^5^ cells/well were seeded and grown until 80% confluence. Each formulation and its isolated components were incubated with and without the predetermined virus titer for 24 h in cell culture. After the washing step with warm PBS, incomplete cell culture medium was added along with 0.5 mg/ml of MTT and incubated for 2 h at 37°C. The absorbance was measured using the microplate reader at 570 nm. Results were performed in triplicates and normalized to the control considered to be 100%.

#### 2.2.5 Formulation Effect in SARS-CoV-2 Titer

Two different treatment approaches were employed: 1) treatment incubation of 24 h, followed by a rinsing step with warm PBS, and then the SARS-CoV-2 infection was executed for another 24 h; 2) incubation of SARS-CoV-2 for 24 h followed by a rinsing step with warm PBS and then the treatment incubation was employed during 24 h. Cell culture supernatant was harvested and submitted to a 3.2.3 process. The non-cytotoxic concentrations of the compounds and formulations were determined as the concentrations that did not lead to more than 50% cell death, as compared to untreated cells.

### 2.3 Gene Expression Under SARS-CoV-2 Infection

#### 2.3.1 Primer and Probe Design

Four sets of primers and probes were designed based on the genome of SARS-CoV-2 (GenBank accession no. MN908947.3) using Primer Express Software (version 3.0.1 Applied Biosystems, Foster, CA, USA). The used primers and probes are identified in **Table 1**.

**Table 1:** Set of primer and probe sequences for the one-step multiplex RT-qPCR.

Gene	Primer forward	Primer reverse	Probe
**Spike**	AAATGATCTCTGCTTTACTAATGTCTATGC	GCAGCCTGTAAAATCATCTGGTAAT	Cy5 – AAGTCAGACAAATCGCTCCAGGGCAAA – BHQ-3
**RdRp** (RNA-dependent RNA polymerase)	GCGGTATGTGGAAAGGTTATGG	AACGATTGTGCATCAGCTGACT	JOE – TTGTGATCAACTCCGCGAACCCATG – TAMRA
**ACE2** (angiotensin converting enzyme 2)	GTGGGAGATGAAGCGAGAGATAG	TGAGTAATCATTAGAAACATGGAACAGA	JOE – CATGATGAAACATACTGTGACCCCGCA – TAMRA
**TMPRSS2** (transmembrane serine protease 2)	CGGACCAAACTTCATCCTTCA	TCCAGTCGTCTTGGCACACA	Cy5 – TGTACTCATCTCAGAGGAAGTCCTGGCACC – BHQ-3
**GAPDH** (glyceraldehyde-3-phosphate dehydrogenase)	TCAAGATCATCAGCAATGCC	TGAGTCCTTCCACGATACC	Cy5 – CCTGCACCACCAACTGCTTAGCAC – BHQ-3

#### 2.3.2 Gene Relative Quantification With the ΔCT Method Using a Reference Gene by One-Step RT-qPCR

Gene expression was estimated measuring the mRNA from cell extraction by RT-qPCR with qTOWER (3) (Analytik Jena, Germany), using One-step NZYSpeedy RT-qPCR Probe Kit, ROX (NZYTech, Lisbon, Portugal). 10 ng/µl RNA was employed, and the threshold cycle (CT) values from each biological assay were plotted with two experimental replicates following the manufacturer’s procedure. Melting curve analysis was used to monitor the specificity of primers and probes. Results were normalized to the GAPDH housekeeping gene, and gene relative expression was employed by the ΔC_T_ expression/ΔC_T_ negative control ratio.

### 2.4 Statistical Analysis

All experiments were performed in triplicate and normalized to a negative control. Statistical analysis was performed comparing the control group results with those of the different groups with two-way analysis of variance (ANOVA) multiple-comparison and Dunnett *post hoc* tests, using GraphPad Prism 8.0.1 software (GraphPad Software, San Diego, CA, USA). Normality of data distribution was assessed using the Shapiro–Wilk test and for the homogeneity of variance with Bartlett’s test. Results were considered statistically significant whenever p-value < 0.05.

## 3 Results and Discussion

### 3.1 The Rationale for Data Analysis

F1T, F2T, and F3T were established using a mixture of specific terpenes purified from *Origanum virens*, *Thymus mastichina*, and *Cannabis sativa* as the genera of those plants are recognized to contain terpenes with reported antimicrobial properties (18, 21, 24–30). The concentration was based on published data and in data from our previous work (results from ecotoxicological and cytotoxic assays to be published, POCI-01-02B7-FEDER-053456-BIOBLOCKCOVID). Moreover, we intended to study the CBD-terpenes’ entourage action (53–55), and so we used a lower concentration of F1T, F2T, and F3T when in combination with CBD. Despite the respiratory tract being the dominant route in SARS-CoV-2 infection, the colon, kidney, and skin comprise COVID-associated symptoms (56–66). Moreover, the colon, kidney, and skin tissues present very considerable levels of ACE2 and TMPRSS2 expression (data obtained from Human Protein Atlas available from http://www.proteinatlas.org, Uhlén M et al., Tissue-based map of the human proteome. Science (2015) PubMed: 25613900 DOI: 10.1126/science.1260419) and hence potential targets for virus infection. We performed the viral reduction assays in several cell lines and quantified ACE2 and TMPRSS2 gene expressions. Both receptors can be considered as targets for SARS-CoV-2, expecting that the coding genes are upregulated. By its turn, it is expected that RdRp and Spike gene expression is upregulated as viral infectivity progress and that gene expression diminishes because of a virucide or antiviral action. As CN2R activation could limit the release of pro-inflammatory cytokines (50) associated with COVID-19, and in this context being a potential therapeutic target for SARS-CoV-2, CBD was included in the formulations (F1TC, F2TC, F3TC) as a partial agonist of CN2R (37–39).

**Figure 1:**
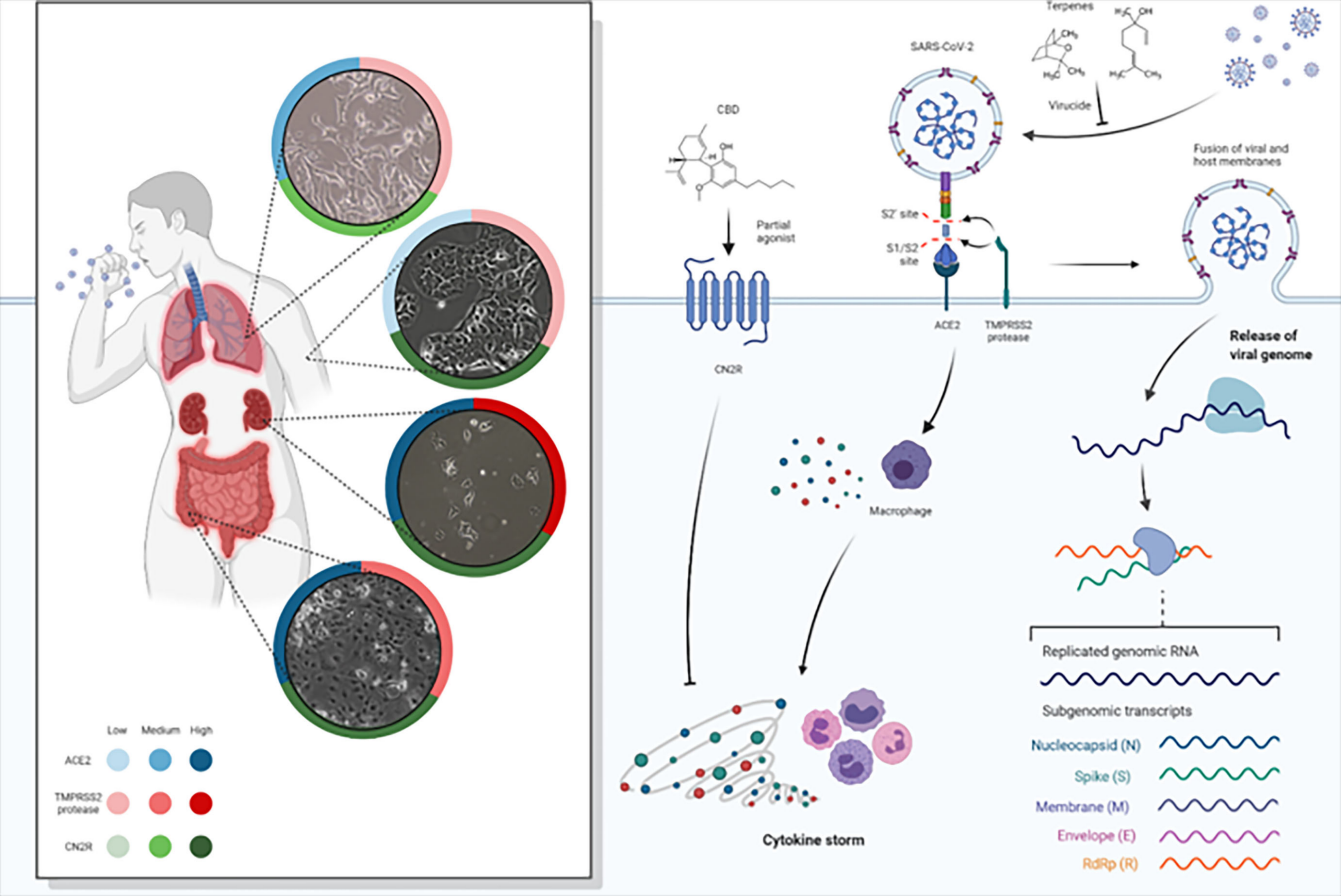
Spike protein of SARS-CoV-2 binds to ACE2 receptors, fusing to the cell membrane and releasing the viral RNA into the host cell. SARC-CoV-2 depends on cellular serine protease, TMPRSS2, for Spike priming. Viral replication in host cells is always associated with inflammation and immune activation being that virus–host–cell interaction produces a set of immune mediators, cytokines, against the virus. It represents the ACE2, TMPRSS2, and CN2R expression levels (low, medium, high) in lung, skin, colon, and kidney tissues (data obtained from Human Protein Atlas available from http://www.proteinatlas.org, Uhlén M et al. Tissue-based map of the human proteome. Science (2015) PubMed: 25613900 DOI: 10.1126/science.1260419). CBD and terpenes (linalool and 1.8-cineole as representative of components of the formulations) are represented as virucide agents blocking and inactivating the virus at an early stage of infection, as antiviral agents blocking the virus cellular machinery and as agents against an overactive immune-inflammatory cascade. As the efficiency of Spike–ACE2 interaction determines SARS-CoV-2 transmissibility, the expression of ACE2 and TMPRSS2 could represent a major risk factor for the susceptibility to SARS-CoV-2 infection. We evaluate the hypothesis of terpenes as virucide agents that could disrupt the interaction between the Spike/TMPRSS2 proteins and the host cell ACE2 receptor. Moreover, after virus entry, terpenes could potentially have an antiviral effect by inhibiting RdRp thus preventing viral replication. Also, it was intended to better understand if a specific group of terpenes and CBD have the potential to act synergistically as therapeutic agents for SARS-CoV-2 and if the action is at early stages or later stages of infection. We assess the hypothesis that CBD may have the potential for modulating the exacerbated inflammatory process typical of COVID-19. CBD is a partial agonist CN2R that is widely expressed in the immune system and, when stimulated, promotes the inhibition of proinflammatory cytokine production, the increase of anti-inflammatory cytokines, and the induction of regulatory T cells.

F1T, F2T, and F3T were established using a mixture of specific terpenes, and the concentration was based on published data and in data from our previous work (results from ecotoxicological and cytotoxic assays to be published, POCI-01-02B7-FEDER-053456-BIOBLOCKCOVID). Moreover, we intended to study the CBD-terpene entourage effect, and so we used a lower concentration of F1T, F2T, and F3T when in combination with CBD. Despite the respiratory tract being the dominant route in SARS-CoV-2 infection, the kidney and colon tissues present very considerable levels of ACE2 and TMPRSS2 expression and hence potential targets for virus infection. We performed the viral reduction assays in several cell lines and quantified ACE2 and TMPRSS2 gene expression. Both receptors can be considered as targets for SARS-CoV-2, expecting that the coding genes are upregulated. By its turn, it is expected that RdRp and Spike gene expression is upregulated as viral infectivity progress and that gene expression diminishes because of a virucide or antiviral action. As CN2R activation could limit the release of pro-inflammatory cytokines associated with COVID-19, and in this context being a therapeutic target for SARS-CoV-2, CBD was included in the formulations (F1TC, F2TC, F3TC) as a partial agonist of CN2R. **Figure 1** illustrates the adsorption and replication mechanisms as well as the potential action of CBD and terpenes and the ACE2, TMPRSS2, and CN2R expression in the lung, skin, colon, and kidney tissues. **Table 2** shows the cytotoxicity evaluation of the formulations per cell line for both pretreatment and treatment conditions, and **Table 3** shows the cytotoxicity of the components that comprises the formulations.

**Table 2:** Cytotoxicity evaluation of formulations.

	Caco-2													
	Control	[F1T.20]	[F1T.100]	[F1TC.10|1]	[F1TC.20|1]	[F2T.20]	[F2T.100]	[F2TC.10|1]	[F2TC.20|1]	[F3T.20]	[F3T.50]	[F3TC.10|1]	[F3TC-20|1]	
NT	100.0 ± 5.2%	112.5 ± 12.0%	109.1 ± 4.1%	97.1 ± 4.7%	98.1 ± 2.9%	116.5 ± 6.7%	107.2 ± 14.4%	104.6 ± 1.0%	98.3 ± 4.8%	91.9 ± 12.9%	*63.1 ± 2.9%*	*5.4 ± 0.8%*	*4.7 ± 0.3%*	CV
	0.0 ± 0.0%	6.6 ± 1.1%	29.6 ± 3.3%	0.0 ± 0.0%	17.5 ± 5.0%	21.9 ± 4.7%	49.0 ± 1.9%	0.0 ± 0.0%	16.9 ± 2.2%	35.1 ± 0.0%	42.3 ± 1.7%	0.0 ± 0.0%	5.3 ± 5.1%	VR
	100.0 ± 4.0%	107.0 ± 8.0%	116.0+0.0%	115.4 ± 8.6%	116.1 ± 5.9%	107.4 ± 0.3%	111.1 ± 0.1%	0.0 ± 0.0%	112.7 ± 6.7%	0.0 ± 0.0%	0.0 ± 0.0%	130.1 ± 0.9%	0.0 ± 0.0%	Ae
	100.0 ± 1.0%	85.3 ± 9.3%	91.3 ± 0.3%	77.6 ± 7.4%	80.3 ± 7.7%	84.6 ± 5.6%	92.6 ± 5.4%	69.9 ± 0.1%	92.3 ± 11.7%	73.7 ± 2.7%	89.3 ± 4.3%	82.4 ± 2.6%	74.4 ± 1.6%	Te
	100.0 ± 5.0%	285.1 ± 2.9%	36.9 ± 0.1%	365.8 ± 12.2%	334.9 ± 6.9%	97.5 ± 1.5%	*281.0 ± 15.0%*	414.0 ± 4.0%	206.1 ± 3.9%	464.1 ± 19.1%	375.1 ± 1.9%	261.9 ± 11.1%	332.0 ± 8.0%	Re
	100.0 ± 1.2%	225.4 ± 3.6%	63.4 ± 2.6%	283.8 ± 7.2%	255.9 ± 7.9%	110.8 ± 5.2%	*222.7 ± 10.7%*	318.8 ± 9.2%	176.2 ± 1.8%	360.3 ± 5.3%	288.1 ± 0.1%	201.2 ± 3.8%	253.3 ± 3.7%	Se
PT	100 ± 4.3%	95.9 ± 10.3%	108.0 ± 4.5%	94.8 ± 7.9%	108.6 ± 8.8%	95.4 ± 2.2%	81.2 ± 0.4%	84.5 ± 8.6%	98.9 ± 4.3%	*9.9 ± 0.4%*	*9.7 ± 0.0%*	*11.5 ± 0.0%*	*11.1 ± 0.3%*	CV
	0.0 ± 0.0%	0.0 ± 0.0%	19.2 ± 5.0%	28.6 ± 3.8%	0.0 ± 0.0%	0.0 ± 0.0%	23.0 ± 3.9%	26.6 ± 1.0%	0.0 ± 0.0%	0.0 ± 0.0%	4.0 ± 3.4%	17.5 ± 2.3%	13.4 ± 3.4%	VR
	100.0 ± 0.5%	113.7 ± 4.3%	126.4 ± 1.4%	116.0 ± 5.0%	141.2 ± 0.8%	124.7 ± 4.3%	109.9 ± 4.1%	125.9 ± 3.1%	136.8 ± 3.2%	0.0 ± 0.0%	0.0 ± 0.0%	0.0 ± 0.0%	0.0 ± 0.0%	Ae
	100.0 ± 2.1%	86.50.5%	108.5 ± 1.5%	97.6 ± 4.4%	80.9 ± 0.1%	83.6 ± 9.6%	79.3 ± 1.3%	99.1 ± 2.9%	95.6 ± 0.6%	105.0 ± 1.0%	85.9 ± 11.1%	96.3 ± 2.7%	71.3 ± 1.3%	Te
	100.0 ± 0.2%	34.4 ± 1.6%	25.4 ± 0.4%	38.4 ± 0.6%	105.7 ± 4.3%	13.9 ± 0.1%	38.1 ± 0.9%	20.4 ± 0.6%	26.0 ± 1.0%	157.1 ± 0.1%	87.1 ± 0.1%	151.1 ± 0.1%	73.6 ± 3.4%	Re
	100.0 ± 2.8%	78.8 ± 3.2%	37.8 ± 0.8%	44.1 ± 5.1%	77.1 ± 7.1%	53.1 ± 5.9%	73.1 ± 5.9%	42.9 ± 8.1%	41.4 ± 4.6%	94.1 ± 6.1	60.1 ± 1.1%	150.1 ± 3.9%	110.5 ± 5.5%	Se
	A549													
	Control	[F1T.20]	[F1T.100]	[F1TC.10|1]	[F1TC.20|1]	[F2T.20]	[F2T.100]	[F2TC.10|1]	[F2TC.20|1]	[F3T.20]	[F3T.50]	[F3TC.10|1]	[F3TC-20|1]	
NT	100.0 ± 5.4%	94.0 ± 7.6%	76.1 ± 3.0%	112.1 ± 19.0%	115.8 ± 6.0%	107.9 ± 20.1%	120.6+80.5%	100.9 ± 5.1%	117.8 ± 9.7%	22.4 ± 0.2%	24.3 ± 1.1%	*22.7 ± 0.4%*	22.2 ± 0.7%	CV
	0.0 ± 0.0%	3.4 ± 0.5%	68.0 ± 4.8%	23.0 ± 0.9%	22.4 ± 0.6%	0.0 ± 0.0%	13.4 ± 4.7%	12.2 ± 0.8%	98.7 ± 5.0%	0.0 ± 0.0%	25.1 ± 0.6%	*96.5 ± 3.0%*	45.0 ± 2.2%	VR
	100.0 ± 7.0%	85.3 ± 1.7%	83.6 ± 0.6%	81.6 ± 4.4%	109.1 ± 0.1%	73.7 ± 4.3%	84.6 ± 3.4%	108.9 ± 7.1%	88.2 ± 0.8%	71.7 ± 4.7%	106.5 ± 0.5%	90.6 ± 1.4%	80.4 ± 3.6%	Ae
	100.0 ± 5.0%	101.2 ± 3.8%	83.3 ± 4.3%	73.1 ± 0.9%	100.6 ± 4.4%	69.1 ± 0.1%	82.8 ± 2.2%	106.0 ± 3.0%	80.4 ± 1.4%	72.5 ± 0.5%	132.9 ± 6.1%	86.0 ± 2.0%	70.4 ± 2.6%	Te
	100.0 ± 3.2%	63.1 ± 2.9%	79.7 ± 2.7%	60.9 ± 0.1%	45.6 ± 0.4%	48.1 ± 3.1%	27.8 ± 1.2%	73.8 ± 3.2%	70.5 ± 4.5%	30.2 ± 0.2%	28.3 ± 1.7%	61.2 ± 0.8%	31.8 ± 1.2%	Re
	100.0 ± 4.4%	59.8 ± 0.2%	76.1 ± 6.1%	66.1 ± 0.9%	44.9 ± 0.9%	51.6 ± 3.6%	33.1 ± 0.1%	75.7 ± 0.3%	73.9 ± 1.1%	24.8 ± 0.8%	39.1 ± 1.9%	60.2 ± 1.2%	43.6 ± 1.4%	Se
PT	100.0 ± 9.4%	105.0 ± 11.6%	84.6 ± 2.6%	95.7 ± 17.6%	60.7 ± 1.1%	77.4 ± 4.1%	80.2 ± 4.2%	89.8 ± 11.5%	63.7 ± 5.7%	6.0 ± 0.3%	6.6 ± 0.3%	24.0 ± 3.3%	5.4 ± 0.1%	CV
	0.0 ± 0.0%	0.0 ± 0.0%	0.0 ± 0.0%	0.0 ± 0.0%	0.0 ± 0.0%	21.9 ± 1.4%	0.0 ± 0.0%?	0.0 ± 0.0%	0.0 ± 0.0%	0.0 ± 0.0%	0.0 ± 0.0%	0.0 ± 0.0%	0.0 ± 0.0%	VR
	100.0 ± 8.0%	106.3 ± 3.7~%	112.0 ± 3.0%	103.1 ± 3.9%	114.4 ± 3.6%	105.4 ± 5.6%	104.0 ± 6.0%	97.5 ± 3.5%	98.5 ± 1.5%	105.8 ± 5.2%	74.1 ± 0.1%	104.8 ± 3.2%	95.0 ± 5.0%	Ae
	100.0 ± 11.1%	121.3 ± 5.7%	124.5 ± 7.5%	113.1 ± 5.9%	128.3 ± 0.7%	125.0 ± 8.0%	115.8 ± 3.2%	109.1 ± 5.9%	118.8 ± 8.2%	121.4 ± 4.4%	74.1 ± 3.9%	110.9 ± 2.1%	99.6 ± 6.4%	Te
	100.0 ± 2.0%	116.2 ± 6.8%	119.6 ± 1.6%	120.7 ± 1.3%	130.4 ± 0.6%	135.6 ± 9.4%	113.5 ± 3.5%	131.6 ± 6.4%	104.8 ± 2.2%	112.4 ± 1.4%	76.7 ± 1.3%	126.9 ± 8.1%	95.4 ± 1.6%	Re
	100.0 ± 2.4%	78.8 ± 3.3%	37.8 ± 0.8%	44.1 ± 5.1%	77.1 ± 7.1%	53.1 ± 5.9%	73.1 ± 5.9%	42.9 ± 8.1%	41.4 ± 4.6%	94.1 ± 6.1%	60.1 ± 1.1%	150.1 ± 3.9%	110.5 ± 5.5%	Se
	HaCaT													
	Control	[F1T.20]	[F1T.100]	[F1TC.10|1]	[F1TC.20|1]	[F2T.20]	[F2T.100]	[F2TC.10|1]	[F2TC.20|1]	[F3T.20]	[F3T.50]	[F3TC.10|1]	[F3TC-20|1]	
NT	100.0 ± 4.7%	90.4 ± 4.1%	105.8 ± 2.6%	118.1 ± 14.1%	105.3 ± 9.0%	109.4 ± 4.7%	92.5 ± 2.6%	124.6 ± 7.6%	102.8 ± 7.7%	*8.0 ± 0.1%*	*8.3 ± 0.6%*	*8.9 ± 0.2%*	*8.7 ± 0.2%*	CV
	0.0 ± 0.0%	26.1 ± 0.6%	31.9 ± 0.5%	19.7 ± 3.7%	37.7 ± 1.9%	30.0 ± 3.6%	46.1 ± 4.8%	18.6 ± 2.8%	23.5 ± 4.4%	43.8 ± 1.0%	*54.9 ± 3.0%*	30.5 ± 4.5%	54.0 ± 4.4%	VR
	100.0 ± 2.0%	136.1 ± 0.9%	134.4 ± 1.4%	168.1 ± 7.9%	143.2 ± 6.8%	139.0 ± 0.0%	160.0 ± 4.0%	124.1 ± 4.9%	0.0 ± 0.0%	151.9 ± 3.9%	120.5 ± 3.5%	0.0 ± 0.0%	0.0 ± 0.0%	Ae
	100.0 ± 1.0%	175.2 ± 7.8%	140.6 ± 7.6%	174.2 ± 0.2%	158.7 ± 5.3%	158.4 ± 2.4%	161.6 ± 1.4%	115.0 ± 5.0%	133.3 ± 2.7%	78.0 ± 8.0%	92.0 ± 2.0%	172.2 ± 5.8%	106.8 ± 3.2%	Te
	100.0 ± 7.3%	*0.4 ± 0.1%*	0.2 ± 0.2%	68.3 ± 1.7%	*0.0 ± 0.0%*	2.2 ± 1.3%	0.0 ± 0.0%	0.2 ± 0.1%	2.7 ± 0.9%	0.0 ± 0.0%	8.5 ± 6.5%	0.0 ± 0.0%	1.6 ± 0.2%	Re
	100.0 ± 5.8%	*3.6 ± 2.4%*	129.5 ± 4.5%	52.1 ± 0.9%	*0.0 ± 0.0%*	4.2 ± 1.3%	0.0 ± 0.0%	6.5 ± 4.5%	0.9 ± 0.1%	0.0 ± 0.0%	9.1 ± 6.9%	3.0 ± 1.4%	14.0 ± 4.0%	Se
PT	100.0 ± 9.3%	109.0 ± 10.0%	130.3 ± 9.6%	145.3 ± 4.0%	103.9 ± 8.4%	124.4 ± 3.3%	137.9 ± 14.7%	141.6 ± 24.2%	136.8 ± 10.6%	*61.0 ± 10.8%*	*19.4 ± 0.3%*	*54.4 ± 5.2%*	*21.1 ± 0.9%*	CV
	0.0 ± 0.0%	0.0 ± 0.0%	0.0 ± 0.0%	0.0 ± 0.0%	0.0 ± 0.0%	0.0 ± 0.0%	0.0 ± 0.0%	0.0 ± 0.0%	0.0 ± 0.0%	0.0 ± 0.0%	0.0 ± 0.0%	0.0 ± 0.0%	0.0 ± 0.0%	VR
	100.0 ± 0.3%	87.4 ± 2.6%	94.4 ± 1.4%	108.6 ± 4.4%	100.9 ± 3.1%	102.8 ± 3.2%	90.0 ± 0.0%	106.3 ± 4.7%	0.0 ± 0.0%	109.3 ± 0.3%	104.9 ± 5.1%	0.0 ± 0.0%	0.0 ± 0.0%	Ae
	100.0 ± 8.2%	118.2 ± 0.8%	138.8 ± 4.8%	103.8 ± 5.2%	133.8 ± 6.2%	109.8 ± 2.2%	80.8 ± 2.2%	100.7 ± 4.3%	91.0 ± 2.0%	61.0 ± 1.0%	88.7 ± 2.3%	99.0 ± 0.1%	91.8 ± 2.2%	Te
	100.0 ± 1.2%	21.3 ± 0.7%	2.2 ± 1.8%	285.7 ± 4.3%	10.4 ± 0.4%	50.2 ± 0.8%	78.0 ± 4.0%	227.5 ± 4.5%	326.1 ± 0.1%	0.8 ± 0.1%	226.5 ± 8.5%	190.3 ± 3.7%	408.2 ± 1.8%	Re
	100.0 ± 2.3%	58.2 ± 1.8%	16.5 ± 0.5%	217.6 ± 10.4%	44.6 ± 0.6%	69.7 ± 1.3%	81.5 ± 4.5%	206.8 ± 5.2%	248.0 ± 12.0%	31.8 ± 0.2%	179.6 ± 4.4%	181.3 ± 3.7%	311.3 ± 7.7%	Se
	Hek293T													
	Control	[F1T.20]	[F1T.100]	[F1TC.10|1]	[F1TC.20|1]	[F2T.20]	[F2T.100]	[F2TC.10|1]	[F2TC.20|1]	[F3T.20]	[F3T.50]	[F3TC.10|1]	[F3TC-20|1]	
NT	100.0 ± 18.2%	167.7 ± 36.4%	95.1 ± 3.1%	141.9 ± 6.8%	156.2 ± 24.5%	179.5 ± 28.7%	155.8 ± 3.0%	170.5 ± 13.2%	155.6 ± 6.5%	*40.0 ± 24.7%*	*22.0 ± 0.8%*	119.4 ± 7.3%?	*37.2 ± 9.2%*	CV
	0.0 ± 0.0%	32.4 ± 3.1%	0.0 ± 0.0%	0.0 ± 0.0%	18.1 ± 7.3%	19.2 ± 2.3%	0.0 ± 0.0%	12.2 ± 1.1%	7.3 ± 1.2%	21.9 ± 1.0%	19.2 ± 0.7%	23.0 ± 2.5%	28.6 ± 0.6%	VR
	100.0 ± 9.5%	69.3 ± 1.7%	101.4 ± 2.4%	104.7 ± 2.3%	92.2 ± 3.8%	61.2 ± 2.2%	105.8 ± 4.2%	74.5 ± 1.5%	92.3 ± 0.3%	90.7 ± 3.7%	110.9 ± 3.1%	103.2 ± 0.8%	88.4 ± 0.3%	Ae
	100.0 ± 3.2%	47.7 ± 0.3%	107.2 ± 5.2%	116.9 ± 5.1%	88.9 ± 4.1%	38.2 ± 0.2%	108.4 ± 3.6%	66.6 ± 1.4%	96.1 ± 0.1%	94.9 ± 1.9%	109.9 ± 2.1%	113.3 ± 1.7%	97.6 ± 4.4%	Te
	100.0 ± 4.0%	59.3 ± 0.7%	153.3 ± 0.7%	114.2 ± 4.8%	119.5 ± 4.5%	59.1 ± 1.9%	58.1 ± 3.1%	116.9 ± 1.1%	121.8 ± 1.2%	194.2 ± 6.2%	166.1 ± 0.1%	140.9 ± 4.1%	166.2 ± 0.8%	Re
	100.0 ± 5.1%	70.1 ± 1.9%	113.5 ± 1.5%	88.9 ± 4.1%	112.8 ± 0.2%	56.9 ± 1.1%	66.3 ± 0.3%	99.2 ± 3.8%	92.9 ± 4.1%	167.3 ± 4.3%	144.0 ± 7.0%	124.3 ± 5.7%	135.3 ± 0.7%	Se
PT	100.0 ± 6.7%	94.9 ± 13.4%	103.9 ± 16.7%	131.2 ± 22.1%	108.0 ± 5.3%	132.6 ± 18.0%	110.3 ± 15.9%	117.7 ± 10.7%	97.5 ± 11.3%	*20.0 ± 0.9%*	*18.6 ± 0.0%*	118.1 ± 2.5%	*19.3 ± 0.3%*	CV
	0.0 ± 0.0%	0.0 ± 0.0%	0.0 ± 0.0%	0.0 ± 0.0%	43.1 ± 5.0%	0.0 ± 0.0%	2.7 ± 0.1%	0.0 ± 0.0%	17.5 ± 3.8%	6.0 ± 2.6%	7.9 ± 0.5%	0.0 ± 0.0%	0.0 ± 0.0%	VR
	100.0 ± 2.0%	83.1 ± 0.1%	86.5 ± 0.5%	90.2 ± 2.8%	87.6 ± 3.4%	92.2 ± 0.2%	80.3 ± 3.7%	78.0 ± 2.0	93.7 ± 2.3%	77.7 ± 3.7%	84.6 ± 3.4%	87.3 ± 1.7%	84.3 ± 3.7%	Ae
	100.0 ± 7.0%	56.2 ± 0.8%	79.7 ± 2.7%	84.6 ± 1.4%	76.1 ± 3.9%	100.8 ± 1.2%	55.9 ± 0.1%	65.6 ± 1.4%	94.7 ± 2.3%	70.8 ± 2.8%	78.6 ± 0.4%	79.4 ± 0.6%	81.8 ± 2.2%	Te
	100.0 ± 5.2%	88.9 ± 3.1%	71.7 ± 1.3%	70.7 ± 1.3%	93.6 ± 2.6%	92.6 ± 3.4%	86.2 ± 0.2%	76.5 ± 3.5%	99.2 ± 0.8%	103.1 ± 0.9%	5.1 ± 0.1%	67.7 ± 0.3%	48.9 ± 2.1%	Re
	100.0 ± 3.7%	97.5 ± 1.5%	70.5 ± 2.5%	72.7 ± 2.3%	91.2 ± 1.2%	83.5 ± 0.5%	87.1 ± 4.1%	78.0 ± 0.0%	91.7 ± 0.3%	105.9 ± 3.1%	9.4 ± 0.6%	67.4 ± 2.6%	51.2 ± 0.8%	Se

CV, cell viability; formulation effect in SARS-CoV-2 titer—VR, viral reduction; and gene relative quantification with the ΔCT method—Ae, ACE expression; Te, TMPRSS2 expression; Re, RdRp expression; Se, Spike expression; NT, normal treatment; PT, pretreatment; F1T, F2T, F3T, terpene formulations comprising a specific group of 7, 8, and 9 terpenes, respectively, in concentrations of 20, 100, or 50 µM; F1TC, F2TC, F3TC, terpene formulations added with CBD to a concentration of 1 µg/ml. In this case, the terpene concentration is 10 or 20 µM.

**Table 3:** Formulation components evaluation regarding Caco-2, A549, HaCaT, and Hek293T cell line viability.

Components |Cell line	Caco-2	A549	HaCaT	Hek293T
**T0.01%/M0.1%/D0.1%**	63.9 ± 4.2%	56.5 ± 10.6%	119.1 ± 16.1%	85.7 ± 1.9%
**T0.001%/M0.1%/D0.1%**	115.5 ± 7.0%	137.8 ± 2.5%	108.4 ± 15.2%	88.5 ± 5.1%
**T0.01%/M0.01%/D0.1%**	51.7 ± 19.1%	73.1 ± 7.6%	112.9 ± 12.9%	78.8 ± 5.4%
**T0.001%/M0.01%/D0.1%**	100.3 ± 9.0%	130.2 ± 7.1%	105.3 ± 11.6%	68.9 ± 7.6%

T, Tween 80; M, MCT; D, DMSO.

A two-way ANOVA analysis was performed to enquire the effect of treatment per cell line type on cytotoxicity, viral reduction, ACE2 expression, TMPRSS2 expression, RdRp expression, and Spike expression. The results of this analysis are presented in **Table 4**. A statistically significant interaction between the normal treatment and the A549 cell line (F (1, 12) = 3.773; p = 0.038) and between the pretreatment and Caco-2 (F (1, 12) = 8.181; p = 0.014), A549 (F (1, 12) = 7.406; p = 0.020), and HaCaT (F (1, 12) = 7,146; p = 0,020) is denoted.

**Table 4:** A two-way ANOVA regarding the effect of treatment per cell line on cytotoxicity, viral reduction, and gene expression.

Treatment	Cell type	Results
Normal treatment	Caco-2	F (1, 12) = 0.424; p = 0.528
	A549	**F (1, 12) = 3.773; p = 0.038**
	HaCaT	F (1, 12) = 1.246; p = 0.306
	HEK293T	F (1, 12) = 1,988; p = 0,159
Pretreatment	Caco-2	**F (1, 12) = 8.181; p = 0.014**
	A549	**F (1, 12) = 7.406; p = 0.020**
	HaCaT	**F (1, 12) = 7,146; p = 0,020**
	HEK293T	F (1, 12) = 3,026; p = 0,108

There was a statistically significant interaction (represented in bold) between the normal treatment and the A549 cell line and between the pretreatment and Caco-2 and HaCaT.

### 3.2 Analysis by Cell Line and per Formulation

#### 3.2.1 Caco-2

F3T is toxic at the concentration of 50 µM. However, F3T is a promising formulation as it reduces viral titer by 35% while cell viability is 91.9%. Interestingly by adding CBD, a much higher toxicity (94.5% of cell death) is promoted; indirectly, this could be the reason why the viral reduction is 0% when using F3T *versus* F3TC. The toxicity is higher in pretreatment. Comparing F1T.100 with F1TC.10|1, in treatment and pretreatment assays it is possible to conclude those formulations as promising as the viral reduction by 29.6% and 28.6%, respectively, and as *Spike* and *RdRp* gene expressions were reduced. Comparing F2T.100 with F2TC.10|1, in treatment and pretreatment assays it was possible to conclude those formulations as promising as the viral reduction by 49.0% and 26.6%, respectively. It is an advantage to use CBD, as the concentration of terpenes, for obtaining approximately the same viral reduction, is ten times lower, and thus it is possible to conclude about an additive effect, which is also denoted when comparing F1T.100 with F1TC.10|1. An interesting fact to be explored is to understand why, comparing F1TC.10|1 pretreatment with F1TC.10|1 or F1TC.20|1 treatment, the *Spike* and *RdRp* gene expression increases. This cell line was used as COVID-19 includes gastrointestinal symptoms, and it remains uncertain if they are caused by direct infection, as aerosol droplets can be swallowed and pass the gastrointestinal tract, or whether they are a consequence of immune system activation.

#### 3.2.2 A549

F3T is toxic at the concentration of 20 µM. Of relevance, regarding treatment assays, F3TC.10|1 reduces viral titer by 96.5% while cell viability is 22.7% and using F3TC.20|1 is less efficient in reducing viral titer. This fact is also denoted in Hek549 pointing out to a critical selection of the terpene concentration to be used when combined with CBD. By comparing F1T with F1TC and F2T with F2TC, in treatment assays, it is possible to conclude about the benefit of adding CBD as the viral titer increases, maintaining the cell viability. F2TC.20|1 is one of the most promising formulations as it reduces the viral titer by 98.7%, the best value obtained, and as expected *Spike and RdRp* gene expression is reduced. The additive effect of CBD is clear, as the concentration of terpenes, for obtaining approximately the same viral reduction, is ten times lower. For this cell line, the pretreatment assays have no impact in viral reduction. This cell line was used as SARS-CoV-2 propagates through aerosol droplets that can be inhaled and infect the upper airways. F2TC.20|1 could be exploited as a promising therapeutic for upper or lower airway infection.

#### 3.2.3 HaCaT

F3T is toxic for the concentration of 20 µM. Pretreatment assays of this cell line are not adequate as viral reduction is 0%. The addition of CBD to F3T has no effect regarding viral reduction or cell viability in this cell line. A comparable effect is denoted regarding F1T.20 vs. F1TC.20|1, in that adding CBD has no (significant) additive effect in viral reduction either in *Spike* or in *RdPp* expression. F2T.20 is the most efficient formulation by reducing by 30% the viral titer and by promoting the downregulation of *Spike* and *RdPp* expression to less than 5%. The addition of CBD to F2T.20 has no additive effect. Importantly, and compared to the other cell lines, in HaCaT a higher expression of *ACE2* and *TMPRSS2* is verified. This cell line was used, as skin lesions and lesions of the vascular system in some SARS-CoV-2-positive patients have been reported. The high levels of *ACE2* and *TMPRSS2* expression could indicate that percutaneous transmission might be a potential risk route for SARS-CoV-2 infection, particularly in conditions of skin dysfunction. Also, the long-term wearing of protective clothing and having contact with disinfectants cause eczematoid dermatitis which might be a risk factor for percutaneous infection. F2T and F1T can be studied as treating skin lesions in SARS-CoV-2 patients. By its turn, F3T.50 could be used for SARS-CoV-2 control in surfaces as it reduces viral titer by 54.9%.

#### 3.2.4 Hek293T

F3T is toxic for almost all the concentrations, except for F3TC.10|1, which is in fact a promising formulation. The addition of CBD to F1T, F2T, or F3T has no effect, regarding viral reduction or cell viability, and promotes upregulation of *Spike* and *RdRp* (F1T and F2T). F1T.20 and F2T.20 are the most promising formulations as the virus titer is 32% and 19%, respectively, and Spike and *RdRp* are downregulated. Similar to the effect in Caco-2, pretreatment and CBD addition is beneficial for this cell line as the virus titer is 43%. Comparing F1T.20 and F2T.20 with F1TC.20|1 and F2T.20|1, it is possible to conclude about a synergistic effect of CBD and terpenes, and that the terpenes from F1T have a higher effect in virus reduction compared to the terpenes from F2T. This cell line was used as COVID-19 includes kidney failure symptoms.

**Figure 2:**
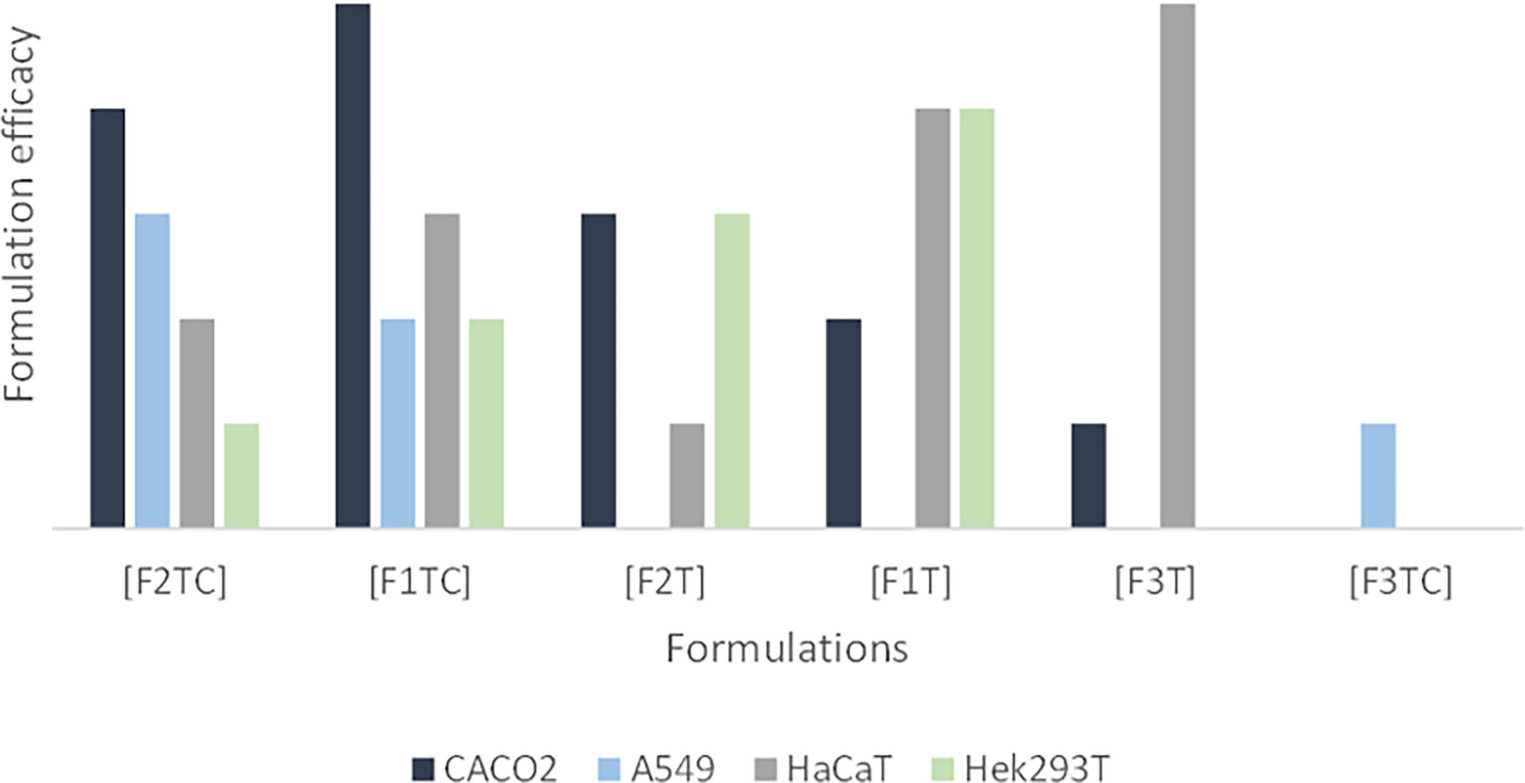
Formulation efficacy (in terms of viral reduction) for Caco-2, A549, HaCaT, and Hek293T cell lines. In this graphic, it is possible to observe i) an additive effect of CBD and terpenes in Caco-2 and A549, ii) that adding CBD to F1T is not advantageous regarding HaCaT, iii) that adding CBD to F2T is an advantage regarding HaCaT, iv) that the terpenes of F1T are more effective than the ones from F2T for HaCaT and Hek293T, vi) that the terpenes from F1T are more effective regarding Caco-2, vii) that F3T and F3TC include terpenes that are more toxic than the ones included in F1T and F2T, being that the toxicity is higher in combination with CBD.

**Table 5** intends to summarize per cell line the most promising formulations based on viral reduction and viral gene expression parameters. In **Figure 2**, it is intended to summarize the efficacy of each formulation per cell line.

**Table 5:** Formulation efficacy considering viral reduction, *RdRp* expression, and *Spike* expression per cell line.

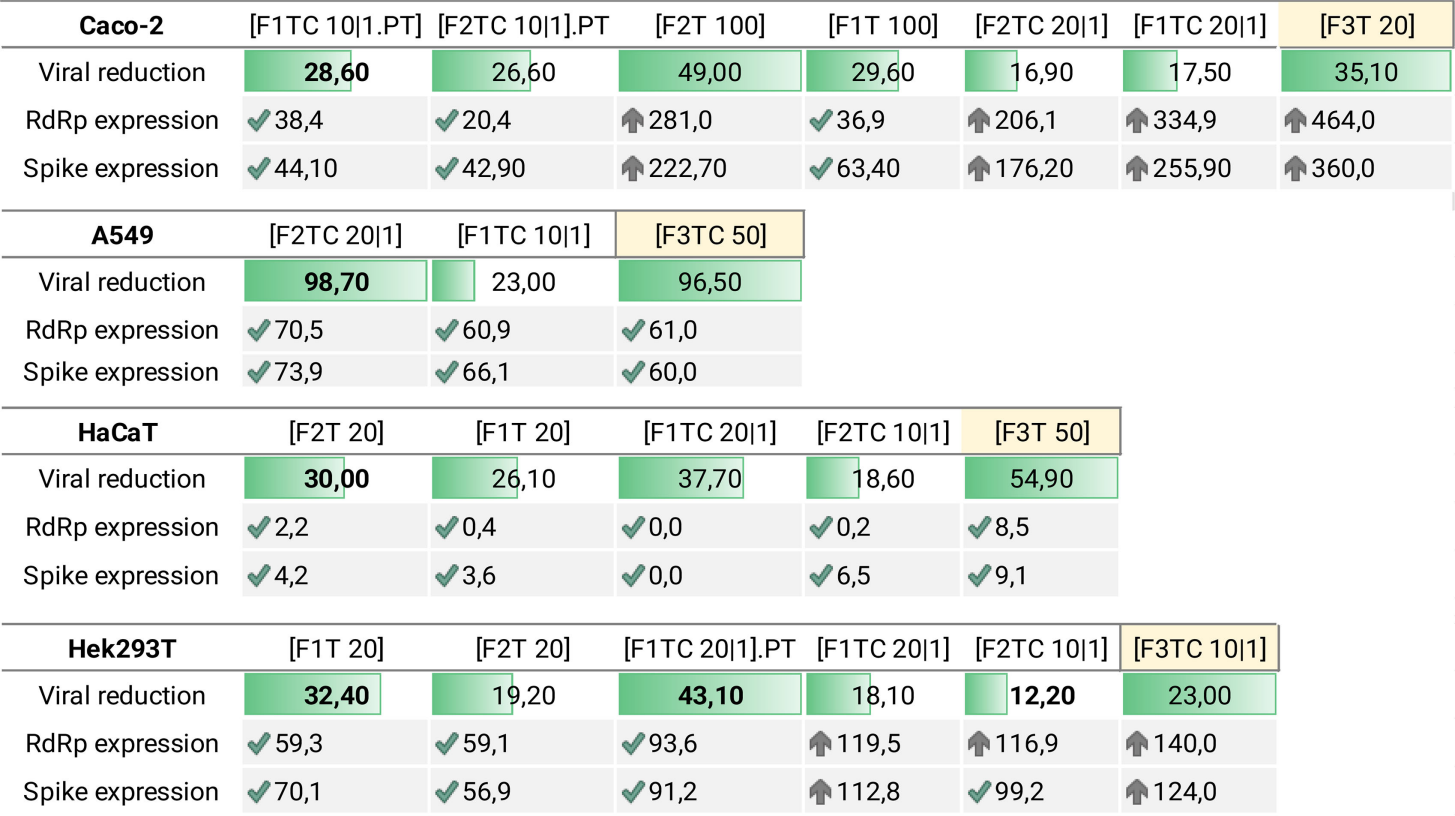	

√—downregulation, ↑—upregulation. PT—pretreatment. The bars indicate the percentage of viral reduction. For Caco-2 and A549, the combination of terpenes with CBD was the most effective treatment.

## 4 Conclusions and Impact

Up to date, a fully effective treatment for COVID-19 is still a challenge. Although the interest in CBD and EOs as therapeutic strategies grows, no scientific studies were made to evaluate the role of CBD and specific terpenes in the progression of SARS-CoV-2 infection. Our group settled an approach focused on the inhibition of both virus entry and viral replication by using biobased formulations from cannabis, thyme, and oregano. The obtained data suggest these formulations to be exploited as new therapeutics targeting COVID-19, providing evidence that CBD and terpenes could be considered for further studies as effective anti-SARS-CoV-2 agents and potentially used for treatment or as adjuvants to conventional COVID-19 therapies. Also, it demonstrates that the selection of terpenes to be used combined with CBD is relevant and points out that treatment should be targeted for afflicted tissues. The proprietary formulations F2TC and F1TC could potentially be used for treating viral infections *via* modulation of the cytokine storm. Additional studies regarding the molecular mechanism explaining both the virucide or antiviral activity and the immunomodulatory effect will be exploited by our group. It will be interesting to explore the anti-inflammatory function of CBD concerning inflammatory events that happen during severe COVID-19 disease and how it might help to prevent the progression from mild to severe disease. In this context, the activation of the ECS could contribute to preventing the progress and the severity of COVID-19. The current study identifies CBD and a specific group of terpenes as a promising anti-COVID-19 therapeutic strategy that warrants further *in vivo* testing and preclinical trials.

## Data Availability Statement

The original contributions presented in the study are included in the article/**Supplementary Material**. Further inquiries can be directed to the corresponding author.

## Author Contributions

All authors certify that they have participated sufficiently in the work to take public responsibility for the content, including participation in the concept, design, analysis, interpretation, writing, or revision of the article. All authors contributed to the article and approved the submitted version.

## Funding

This research was partially funded by the European Commission, European Regional Development, FEDER/02/SAICT/2020/072560 SI-B7-2020-15, POCI-01-02B7-FEDER-053456, BIOBLOCKCOVID. The funding allowed to perform the collection and harvesting of the medicinal plants, extract and purify the terpenes and formulations, execute the formulations, and execute preliminary assays related to toxicity. Considering *in vitro* virucide assays and gene expression assays, the work was partially funded by FCT – Fundação para a Ciência e Tecnologia (REF UID/BIM/04293/2019) and was partially supported by grants 104 and 112 of the 1st edition of RESEARCH4COVID (FCT) and by the grant 418 from the 2nd edition of RESEARCH4COVID-19 (FCT). This work was also partially supported by FEDER-European Regional Development Fund with the grant FEDER/02/SAICT/2020/072560.

## Conflict of Interest

Authors SS, AC, IL, SR and MM was employed by company EXMceuticals Portugal Lda.

​​​​​The remaining authors declare that the research was conducted in the absence of any commercial or financial relationships that could be construed as a potential conflict of interest.

## Publisher’s Note

All claims expressed in this article are solely those of the authors and do not necessarily represent those of their affiliated organizations, or those of the publisher, the editors and the reviewers. Any product that may be evaluated in this article, or claim that may be made by its manufacturer, is not guaranteed or endorsed by the publisher.

## Supplementary Material

Supplementary Figure 1
**–** GC-MS quantitative chromatogram of formulations F1T **(A)**, F2T **(B)** and F3T **(C)**. Peak numbering represents terpenes ranked from the higher to the lower concentration. X axis - Retention Time (min), Y axis – Intensity. The terpenes are anonymized as F1T, F2T, F3T are proprietary formulations. HPLC-UV chromatogram analysis of a CBD isolate **(D)**, X axis - Retention Time (min), Y axis - Intensity (mAU).

Supplementary Figure 2
**–** Graphical representation of the cytotoxicity evaluation of formulations, formulation effect in SARS-CoV-2 titer and gene relative quantification with ΔCT method. Evaluation of the effect from formulations without (F1T=1, F2T=2, F3T=3) and with the addition of CBD (F1TC=1+, F2TC=2+, F3TC=3+). The terpenes were used in a concentration of 20 µM (L) and 100 µM (H) for F1T and F2T, 20 µM (L) and 50 µM (H) for F3T, 10 µM (L) and 20 µM (H) for F1TC and F2TC, 20 µM (H) and 10 µM (L) for F3TC. **(A)** Cytotoxicity effect; **(B)** Formulation effect on viral number copies reduction; **(C and D)** Gene expression of ACE2 and TMPRSS2 genes under the influence of the formulations; **(E and F)** Gene expression of RdRp and Spike genes under the influence of the formulations. Results are represented as mean with standard deviation and normalized to GAPDH expression. Symbols above bars represent statistical significance (* p < 0.05; ** p < 0.01; *** p < 0.001; **** p < 0.0001). **Figure 2** **.1 –** Treatments administrated after SARS-CoV-2 infection in Caco-2 cell line. **Figure 2** **.2 -** Treatments administrated before SARS-CoV-2 infection in Caco-2 cell line. **Figure 2** **.3 -** Treatments administrated after SARS-CoV-2 infection in A549 cell line. **Figure 2** **.4 –**Treatments administrated before SARS-CoV-2 infection in A549 cell line. **Figure 2** **.5 –** Treatments administrated after SARS-CoV-2 infection in HaCaT cell line. **Figure 2** **.6 -** Treatments administrated before SARS-CoV-2 infection in HaCaT cell line. **Figure 2** **.7 -** Treatments administrated after SARS-CoV-2 infection in Hek293T cell line. **Figure 2** **.8 –**Treatments administrated before SARS-CoV-2 infection in Hek293T cell line.

## Abbreviations

ACE-2, angiotensin-converting enzyme 2; THC, tetrahydrocannabinol; ECS, endocannabinoid system; CNR2, cannabinoid receptor 2; RSV, respiratory syncytial virus; EOs, essential oils; HIV, human immunodeficiency virus; MCT, medium-chain triglyceride; CBD, cannabidiol; CPC, centrifuge partition chromatography; GC-MS, gas chromatography-mass spectrometry; HPLC, high-performance liquid chromatography; RdRp, RNA-dependent RNA polymerase; SARS-CoV, severe acute respiratory syndrome-CoV; SARS-CoV-2, severe acute respiratory syndrome-CoV-2.
