# Supplementary figures and images for: Cannabidiol and Terpene Formulation Reducing SARS-CoV-2 Infectivity Tackling a Therapeutic Strategy
Source: Front Immunol. 2022 Feb 15;13:841459. doi: 10.3389/fimmu.2022.841459 (PMC8886108; doi:10.3389/fimmu.2022.841459)

A

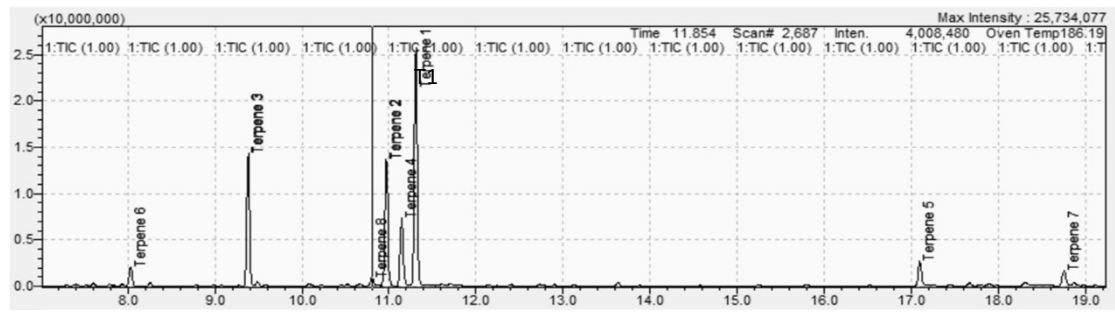

B

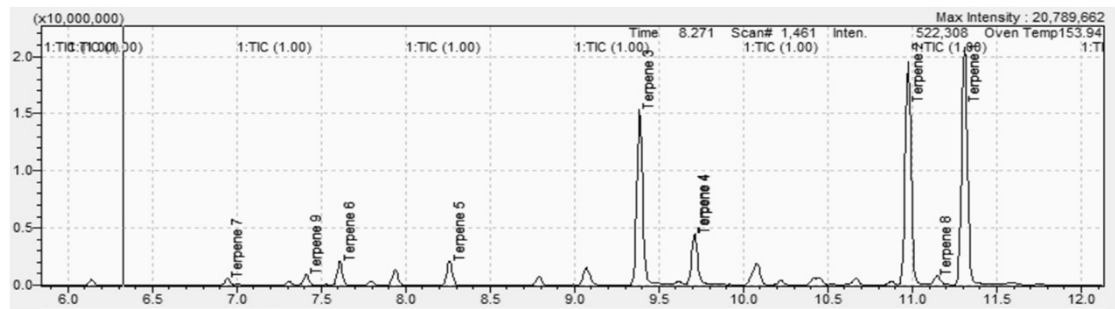

C

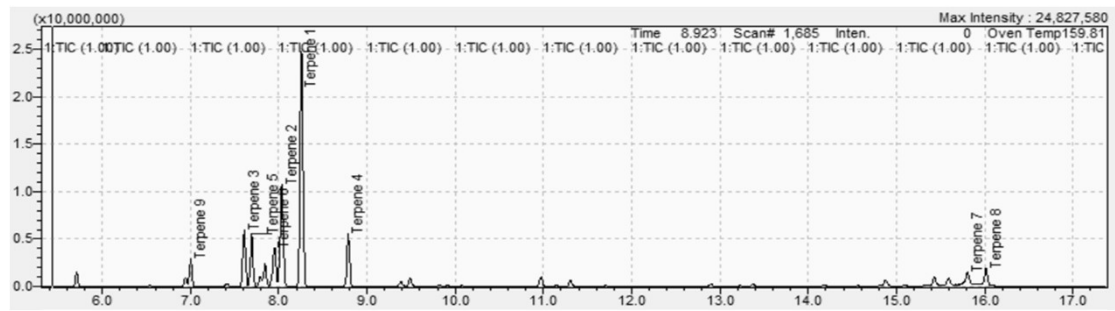

D

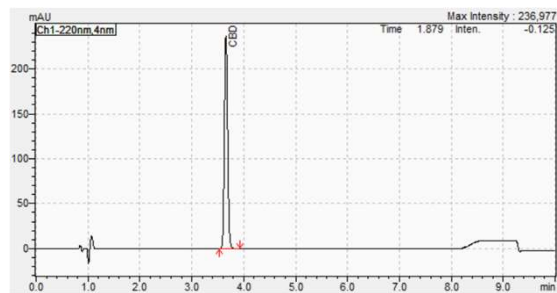

Supplement: Supplementary Figure 1 — – GC-MS quantitative chromatogram of formulations F1T (A), F2T (B) and F3T (C). Peak numbering represents terpenes ranked from the higher to the lower concentration. X axis - Retention Time (min), Y axis – Intensity. The terpenes are anonymized as F1T, F2T, F3T are proprietary formulations. HPLC-UV chromatogram analysis of a CBD isolate (D), X axis - Retention Time (min), Y axis - Intensity (mAU). [file DataSheet_1.pdf]
