# Supplementary material for: Cannabidiol and Terpene Formulation Reducing SARS-CoV-2 Infectivity Tackling a Therapeutic Strategy
Source: Front Immunol. 2022 Feb 15;13:841459. doi: 10.3389/fimmu.2022.841459 (PMC8886108; doi:10.3389/fimmu.2022.841459)
Supplement: Supplementary Figure 2 — – Graphical representation of the cytotoxicity evaluation of formulations, formulation effect in SARS-CoV-2 titer and gene relative quantification with ΔCT method. Evaluation of the effect from formulations without (F1T=1, F2T=2, F3T=3) and with the addition of CBD (F1TC=1+, F2TC=2+, F3TC=3+). The terpenes were used in a concentration of 20 µM (L) and 100 µM (H) for F1T and F2T, 20 µM (L) and 50 µM (H) for F3T, 10 µM (L) and 20 µM (H) for F1TC and F2TC, 20 µM (H) and 10 µM (L) for F3TC. (A) Cytotoxicity effect; (B) Formulation effect on viral number copies reduction; (C and D) Gene expression of ACE2 and TMPRSS2 genes under the influence of the formulations; (E and F) Gene expression of RdRp and Spike genes under the influence of the formulations. Results are represented as mean with standard deviation and normalized to GAPDH expression. Symbols above bars represent statistical significance (* p < 0.05; ** p < 0.01; *** p < 0.001; **** p < 0.0001). Figure 2 .1 – Treatments administrated after SARS-CoV-2 infection in Caco-2 cell line. Figure 2 .2 - Treatments administrated before SARS-CoV-2 infection in Caco-2 cell line. Figure 2 .3 - Treatments administrated after SARS-CoV-2 infection in A549 cell line. Figure 2 .4 –Treatments administrated before SARS-CoV-2 infection in A549 cell line. Figure 2 .5 – Treatments administrated after SARS-CoV-2 infection in HaCaT cell line. Figure 2 .6 - Treatments administrated before SARS-CoV-2 infection in HaCaT cell line. Figure 2 .7 - Treatments administrated after SARS-CoV-2 infection in Hek293T cell line. Figure 2 .8 –Treatments administrated before SARS-CoV-2 infection in Hek293T cell line. [file DataSheet_2.pdf]

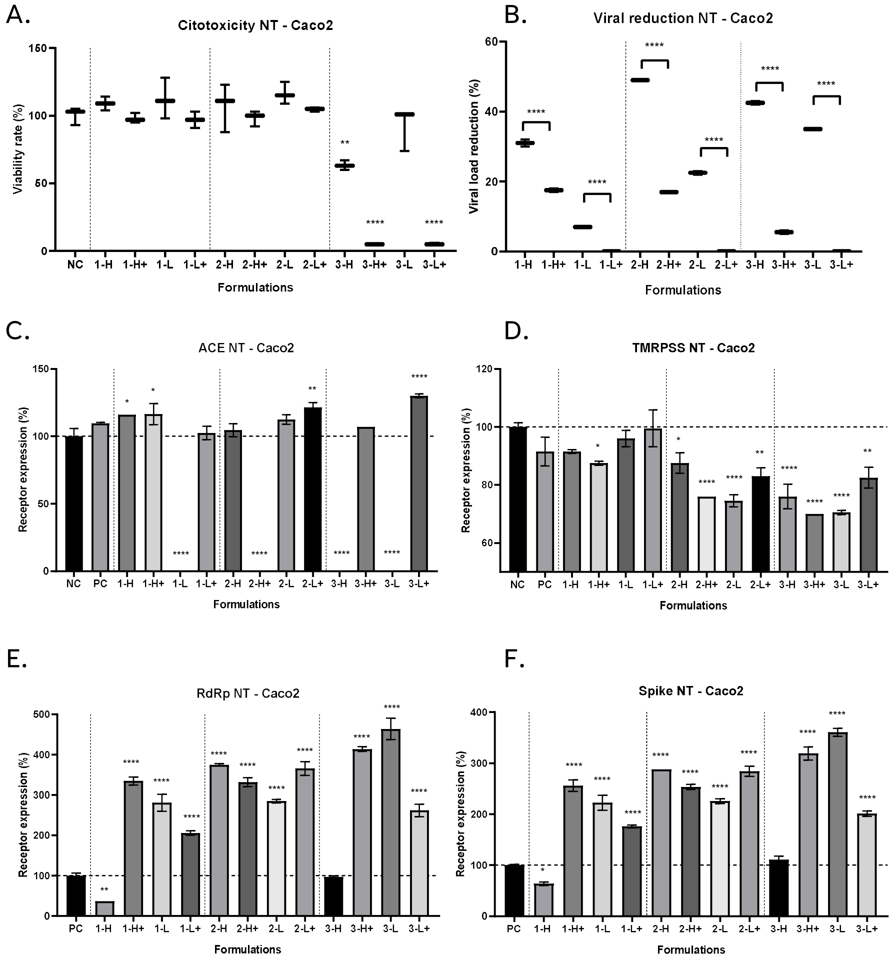


FIGURE 2.1 – CACO2 TREATMENT

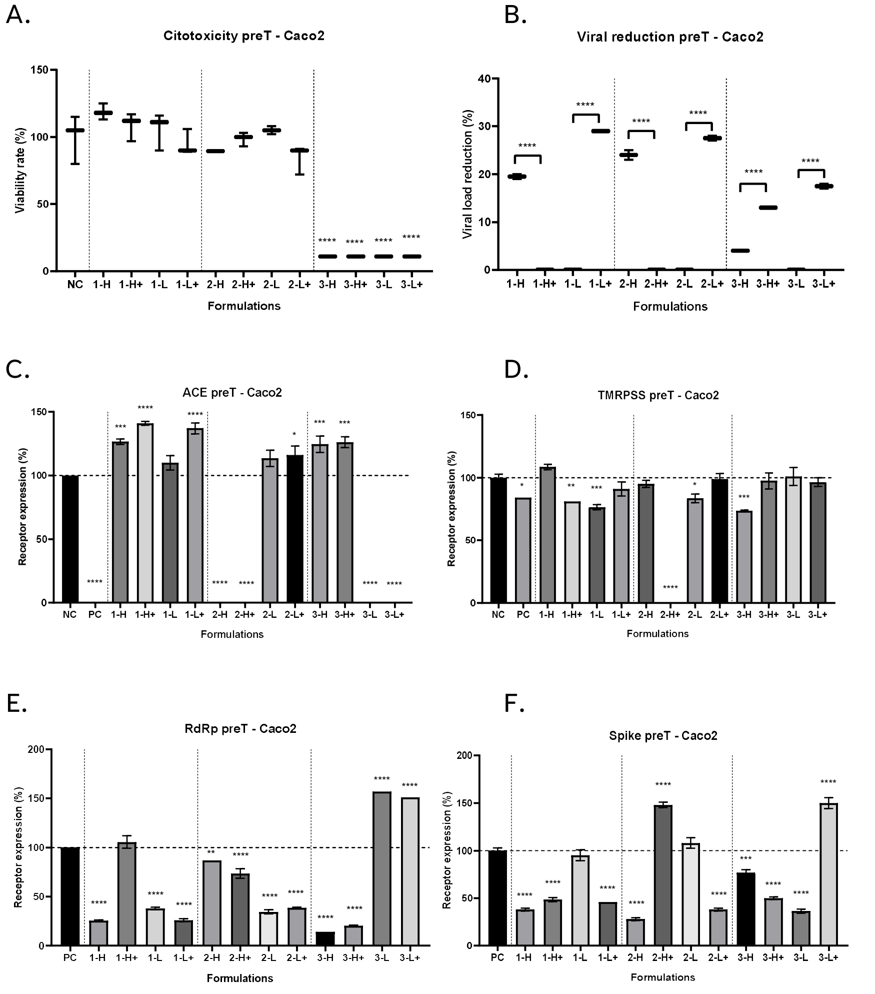


FIGURE 2.2 – CACO2 PRETREATMENT

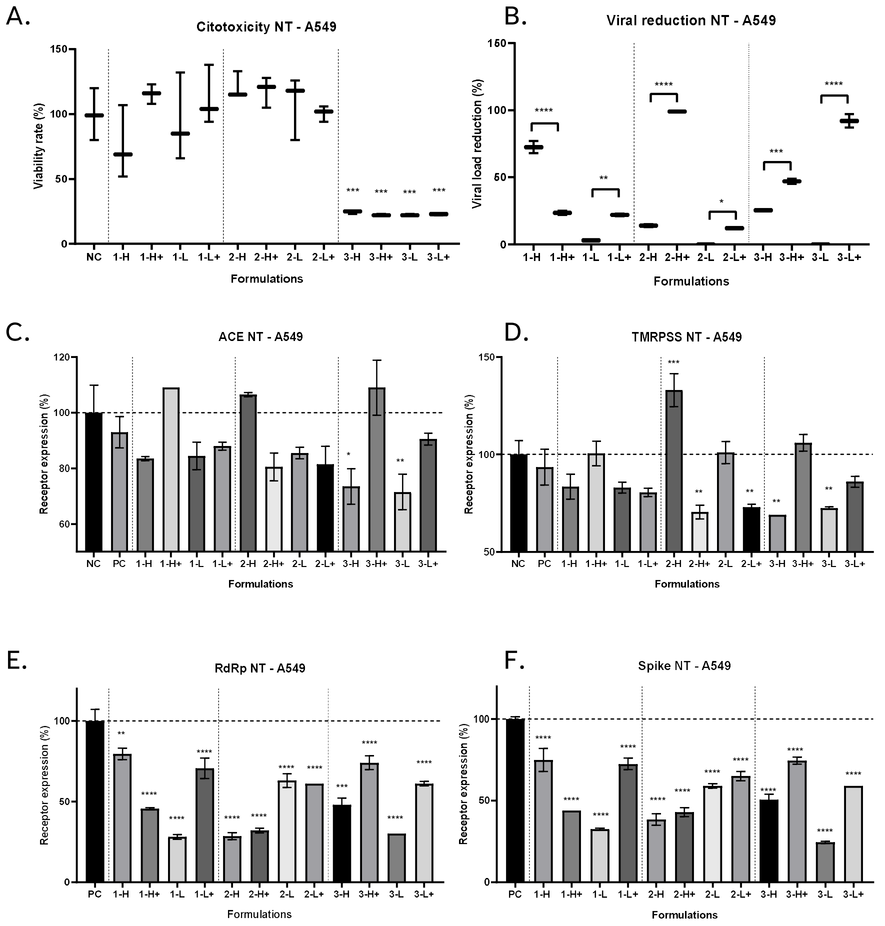


FIGURE 2.3 – A549 TREATMENT

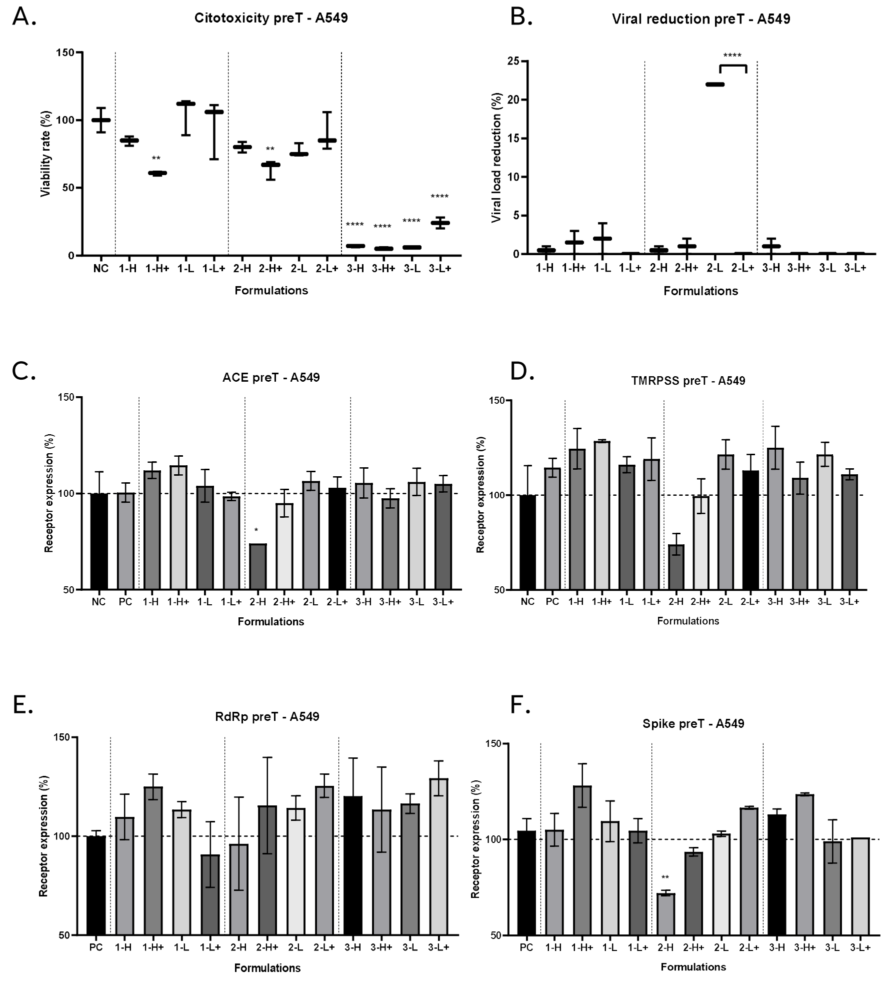


FIGURE 2.4 – A549 PRETREATMENT

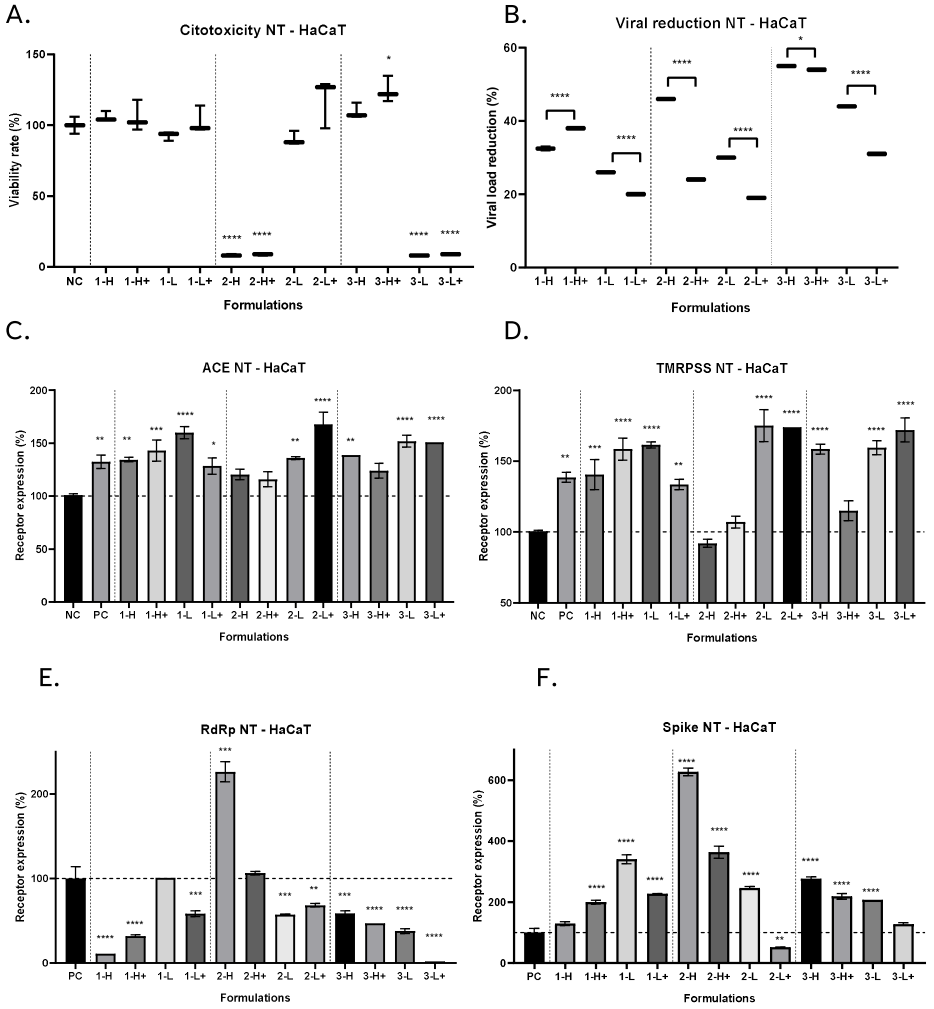


FIGURE 2.5 – HACAT TREATMENT

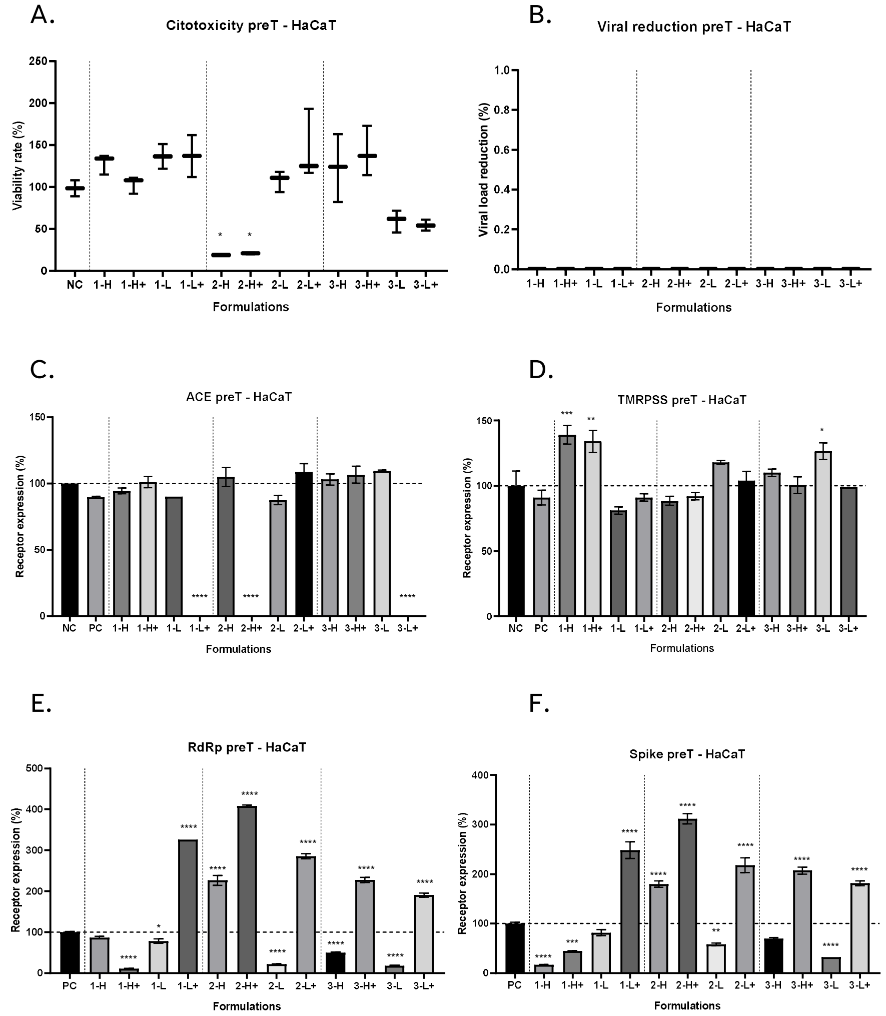


FIGURE 2.6 – HACAT PRETREATMENT

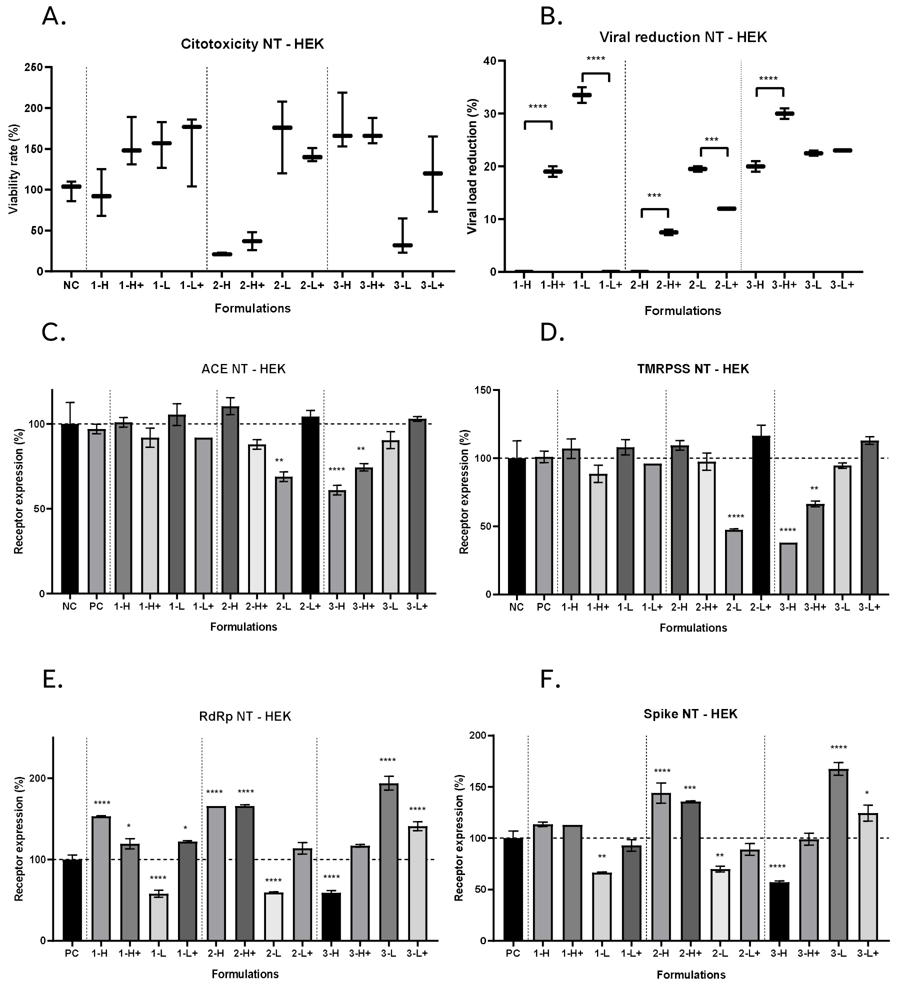


FIGURE 2.7 – HEK293T TREATMENT

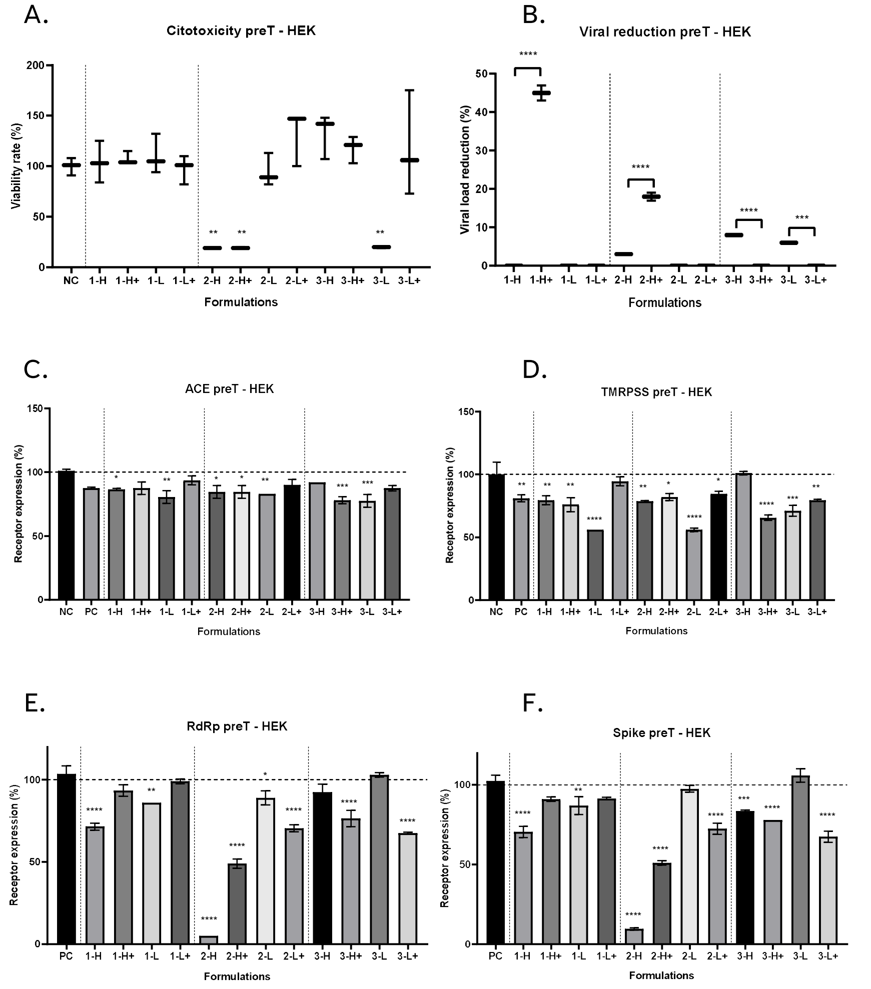


FIGURE 2.8 – HEK293T PRETREATMENT
